# Integrative and Complementary Practices in the Unified Health System: progresses and challenges

**DOI:** 10.1590/1518-8345.2854.3041

**Published:** 2018-09-17

**Authors:** Leandra Andréia de Sousa, Nelson Filice de Barros

**Affiliations:** 1 PhD, Researcher, Faculdade de Ciências Médicas, Universidade Estadual de Campinas, Campinas, SP, Brazil.; 2 PhD, Assistant Professor, Faculdade de Ciências Médicas, Universidade Estadual de Campinas, Campinas, SP, Brazil.

The article by Melo et al.^1^, published in the volume 26 of this journal showed that music therapy, one of the Integrative and Complementary Practices (PIC - *Práticas Integrativas e Complementares*) recently implemented in the Brazilian Unified Health System (SUS - *Sistema Único de Saúde*)^2^, was significant in the reduction of anxiety in chronic patients. Regarding the nursing care with music therapy, the amount of research is rather limited; however, the use of music therapy in Nursing, as well as other PIC, have been encouraged both nationally and internationally^1^ ^,^ ^3^.

Despite the local and global recommendation to offer PIC in national health systems^4^, the topic is still quite controversial, with defenders and detractors. In Brazil, since 1985, documents, resolutions and events marked the regulation process of those practices, and, in 2006, after an intense effort of different individual and institutional agents, the National Policy on Integrative and Complementary Practices (PNPIC - *Política Nacional de Práticas Integrativas e Complementares*)^5^ was published. Before its publication, a situational diagnosis was conducted on the PIC offered by SUS in Brazilian municipalities to identify the most common and viable practices. Thus, in 2006, the Homeopathic Medical Rationality, the Traditional Chinese/Acupuncture Medical Rationality, the Anthroposophical Medical Rationality, Phytotherapy/medicinal plants and Thermalism became part of SUS.

Throughout those 12 years, the institutionalization process of PNPIC was hampered by the lack of an official political national coordination in the Ministry of Health and by the lack of budget allocation to its deployment and implementation. On March 12, 2018, the Minister of Health created the national coordination of PNPIC under the Primary Health Care Directorate of the Ministry of Health. It is a historical achievement, although there is little to celebrate, as until now the national coordination was not regulated and there are many information gaps about, for example, the process of inclusion of fourteen Practices in March 2017^2^ and the inclusion of other 10 Practices in March 2018.

It is known that data from the National Primary Care Access and Quality Improvement Program (PMAQ - *Programa Nacional de Melhoria do Acesso e de Qualidade da Atenção Básica*) are mentioned to justify the implantation of those 24 practices; however, there is no new policy in the current edition of PNPIC^5^ ^)^ that details the recommended criteria in the *Estrategia de la OMS sobre medicina tradicional 2014-2023* ^4^ ^).^ The challenges of implementing PIC in health systems are not exclusively Brazilian, and a research performed in 39 European countries showed that 70% of them do not have specific regulation for PIC^6^.

The commitment to the therapeutic plurality of SUS and its safe, effective, and quality offer, lead us to question the form and content of the implementation of new practices in SUS. The alleged recent progress of PNPIC seems more like a threat to PIC and, in this context coated with challenges, it is observed that the political and scientific need to discuss the ingoing implementation and the relevance of PIC as a integrative care model in SUS.
